# Preliminary Evaluation of the Sural Nerve Using 22-MHz Ultrasound: A New Approach for Evaluation of Diabetic Cutaneous Neuropathy

**DOI:** 10.1371/journal.pone.0032730

**Published:** 2012-04-27

**Authors:** Fang Liu, Jiaan Zhu, Mei Wei, Yuqian Bao, Bing Hu

**Affiliations:** 1 Department of Ultrasound, Shanghai Jiao Tong University Affiliated Sixth People’s Hospital; Shanghai Institute of Ultrasound in Medicine, Shanghai, China; 2 Department of Endocrinology and Metabolism, Shanghai Diabetes Institute, Shanghai Jiao Tong University Affiliated Sixth People’s Hospital, Shanghai, China; The University of Hong Kong, Hong Kong

## Abstract

**Background:**

The application of 22-MHz high-frequency ultrasound allows for visualization of the inner part of the sural nerve. The aim of this study was to evaluate the morphological changes of sural nerves in patients with type 2 diabetes mellitus using ultrasound.

**Materials and Methods:**

The thickness/width (T/W) ratio, the cross-sectional area (CSA) of the sural nerves and the maximum thickness (MT) of the nerve fascicles were measured in 100 patients with type 2 diabetes mellitus and 50 healthy volunteers using 22-MHz ultrasound. Receiver operating characteristic (ROC) curves were plotted to determine the optimal cut-off values as well as the sensitivities and specificities. All parameters were significantly different between the subject and control groups. The ROC curves demonstrated that the MT was the most predictive of diabetic cutaneous neuropathy, with an optimal cut-off value of 0.365 mm that yielded a sensitivity of 90.3% and a specificity of 87.7%.

**Conclusions:**

The results of this study suggest that 22-MHz ultrasound may be a valuable tool for evaluating diabetic cutaneous nerve neuropathy.

## Introduction

The most prevalent type of diabetic peripheral neuropathy (DPN) is distal symmetric, peripheral polyneuropathy, which is primarily sensory in nature [1]. The initial effect of the degenerative process is thought to occur in the cutaneous nerve fiber endings [2]. The diagnosis of diabetic cutaneous neuropathy is based primarily on clinical examination and nerve conduction studies (NCS). However, studies focusing on monitoring the morphological changes of diabetic cutaneous nerves by ultrasound (US) have not been previously performed. The newer 22-MHz high-frequency US allows for visualization of the inner part of the sural nerve (SN). The aim of this study was to evaluate the morphological changes of SNs in patients with type 2 diabetes mellitus using 22-MHz high-frequency US.

## Materials and Methods

The study was approved by the ethics committee of the Shanghai Jiaotong University Affiliated Sixth People’s Hospital, and written consent was obtained from all participants.

This was a prospective study, consisting of subjects consisted of 100 consecutive patients with type 2 diabetes and 50 healthy volunteers as controls. The diabetic patients were divided into a neuropathic group and a non-neuropathic group. Diabetic neuropathy was diagnosed if one or two of the following criteria were present: 1) the Diabetic Neuropathy Symptom (DNS) score was 1 or higher, which was considered positive [3] and 2) routine motor and sensory NCS had been performed, as suggested previously by Fioretti and Ziegler [4], [5]. The control group consisted of 50 healthy volunteers who did not have neuropathic symptoms or any risk factors for neuropathy based on a neurological examination.

**Figure 1:**
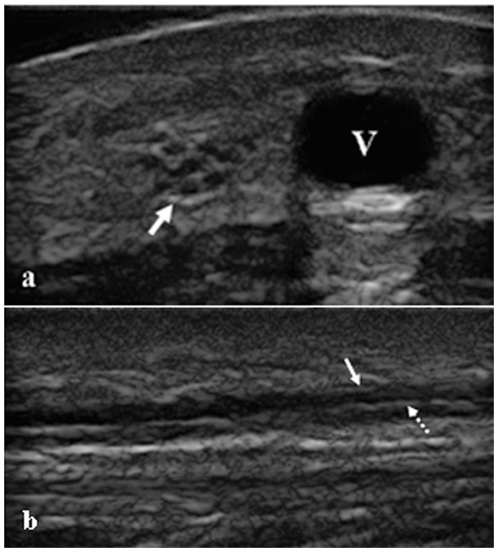
Transverse and longitudinal sonograms of the SN. a) Transverse sonogram of the SN. A transverse sonogram showing the SN (arrows) near the lesser saphenous vein (V) SN appeared as an ovoid hypoechoic reticular structure with a hyperechoic rim. b) Longitudinal sonogram of the SN. A Longitudinal sonogram of the SN showing the perineurium (dashed arrows) and epineurium (solid arrow).

Sonographic examinations were performed with 22-MHz linear-array transducers connected to a MyLabSat (Esaote Biomedica, Genoa, Italy). The sonographer was blinded to the NCS results. All of the subjects were in the prone position during the exam. The transducer was placed in a transverse position on the lower section of the calf. To make sure the sural nerve was not compressed, the operator exerted minimal pressure on the skin and any compression that could cause deformation of the lesser saphenous vein was avoided. On the transverse sonograms, the SN usually appeared as an ovoid hypoechoic reticular structure with a hyperechoic rim near the lesser saphenous vein (Fig. 1a). On the longitudinal sonograms, the SN appeared as multiple hypoechoic bands corresponded to neuronal fascicles, which were separated by hyperechoic lines that correspond to the epineurium (Fig. 1b).

**Figure 2:**
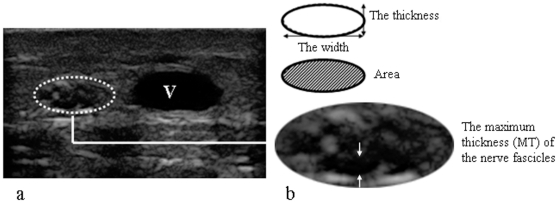
Image analysis on T/W ratio, CSA and MT of the SN. a) Transverse sonogram of the SN. b) Schematic diagram associated with the measurements.

The thickness divided by width on the transverse image of the SN was defined as the thickness/width (T/W) ratio, which is an indicator of the level of swelling and deformation of the SN (Fig. 2). The cross-sectional areas (CSAs) of the SN, which were traced along the outer hyperechoic rim on the transverse sonograms, were calculated using the formula for an ellipse after determination of six optional points along the nerve (Fig. 2). The maximum thickness (MT) of the nerve fascicles was measured along the short axes (Fig. 2). However, there are some anatomical variants of the SN [6], [7]. The classic pattern of sural nerve formation is the union of the medial sural cutaneous nerve (MSCN) and the lateral sural cutaneous nerve (LSCN). The union of the MSCN and LSCN occurred most frequently in the distal third of the leg [8]. Therefore, we chose a location approximately 5 cm proximal to the lateral malleolus as the measurement site. Each SN was measured three times, and the mean value was used in further calculations. Intra-observer reliability was evaluated in 30 cases that were selected by another radiologist.

**Table 1:** Ultrasound-detected anatomical types of SNs.

Type of SN	Control group		Non-neuropathic group		Neuropathic group		Total	
	n	%	n	%	n	%	n	%
I	85	85	94	87	76	83	255	85
II	14	14	13	12	15	16	42	14
III	1	1	1	1	1	1	3	1
Total	100	100	108	100	92	100	300	100

**Table 2:** Comparison and statistical significance of the ultrasonographic parameters.

	Control group	Non-neuropathic group	Neuropathic group	*p*
CSA(mm^2^)	1.44±0.34	1.52±0.35	1.88±0.50	0.024
MT(mm)	0.33±0.04	0.36±0.08	0.45±0.07	<.001
T/W ratio	0.51±0.07	0.56±0.10	0.59±0.09	0.002

Abbreviations: CSA, cross-sectional area; MT, the maximum thickness of the neuronal fascicles; T/W ratio, thickness/width ratio. All values in the three groups are the mean±standard deviation.

The results of the statistical analysis are presented as the mean±SD, and a P-value of less than 0.05 was considered statistically significant. The one-way ANOVA was used to compare the groups. The intra-observer reliability was tested with the intra-class correlation coefficient (ICC). Receiver operating characteristic (ROC) curves were employed to determine the optimal cut-off values for useful ultrasonographic variables.

**Figure 3:**
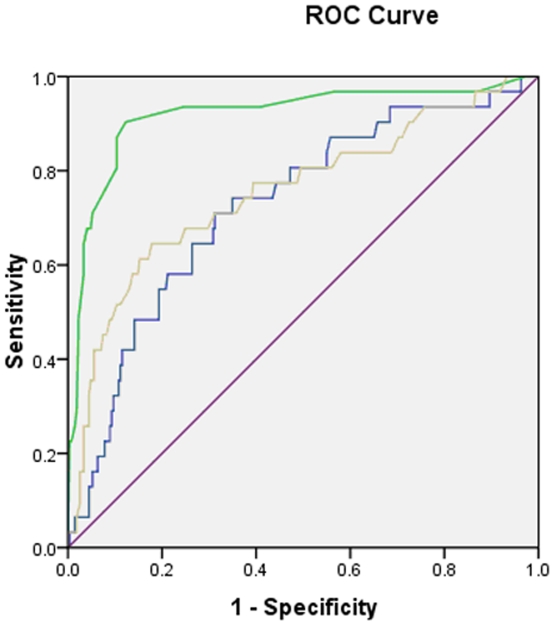
Receiver operating characteristic (ROC) curve of the US parameters. The green curve represents the maximum thickness of the neuronal fascicles (MT), the yellow curve represents the cross-sectional area (CSA), and the blue curve represents the thickness/width (T/W) ratio.

## Results

A total of 92 SNs from the neuropathic group (21 males and 25 females; mean age, 65.1±10.2 years; BMI, 24.3±4.7), 108 SNs from the non-neuropathic group (28 males and 26 females; mean age, 63.2±9.8 years; BMI, 23.5±3.9), and 100 SNs from the control group (25 males and 25 females; mean age, 62.5±13.7 years; BMI, 24.7±2.8) were examined using US. There were no statistically significant differences in age or BMI between the groups; however, there was a statistically significant difference in the duration of diabetes between the neuropathic group (7.5±2.6 years) and the non-neuropathic group (3.9±1.8 years).

The inner part of the SN, consisting of the fascicle, perineurium and epineurium, was identified in all participants. The ICCs for the CSA, T/W ratio and MT were 0.810, 0.879 and 0.912, respectively (*p*<0.001). The types of SN were shown in Table 1.The mean values and standard deviations (SD) of these parameters along with the statistical significance of the differences between the control group, the non-neuropathic group and the neuropathic group are listed in Table 2. There was a statistically significant difference between the three groups regarding all US parameters investigated.

The ROC (Fig.3) curve of the MT revealed that the area under the curve (AUC) was 0.918 (*p*<0.001) with an optimal cut-off value of 0.365 mm, yielding a sensitivity of 90.3% and a specificity of 87.7%. For the CSA, the ROC curve demonstrated that the AUC was 0.755 (*p<*0.001) with an optimal cut-off value of 1.685 mm^2^, resulting in a 64.5% sensitivity and an 82.2% specificity. Furthermore, the ROC curve of the T/W ratio showed that the AUC was 0.726 (*p*<0.001) with an optimal cut-off value of 0.528, yielding a 71% sensitivity and a 68.8% specificity. The MT of SN had the best diagnostic accuracy.

## Discussion

DPN is an important chronic complication of diabetes. Worsening neuropathy is accompanied by a increasing loss of sensory nerve fibers. When the fibers undergo degeneration or impaired remyelination, they release impulses of positive symptoms. With progression of the disease, the negative symptoms of sensory loss increased [9]. Clinical examination and NCS are used to support the diagnosis of diabetic cutaneous neuropathy. A growing number of researchers now believe that it is important to identify subclinical neuropathy before the clinical findings appear because patients may respond better to treatment if the condition is diagnosed early [10]. However, NCS also has limitations and disadvantages. Its application is painful and it cannot assess the sural nerve anatomy. Therefore, there continues to be controversy about the sensitivity and specificity of NCS for the diagnosis of subclinical neuropathy [1].

This study demonstrates that 22-MHz high-frequency US is a painless diagnostic technique that can be used to evaluate morphological changes in the SNs of diabetic patients. Because the inner parts of the SN could be clearly identified, the morphological parameters could be accurately measured.

The SN is commonly used to study distal sensory polyneuropathy in diabetic patients. However, there are some anatomical variants of the SN. We classified the SN into three types using US [11]. Type I was the classic pattern of sural nerve formation, in which both the MSCN and the LSCN contributed to the formation of the SN. Type II lacked the LSCN, whereas type III lacked the MSCN. In this study, type I was more common (85%) than the other types. Furthermore, in type I, the union of the MSCN and LSCN occurred most frequently in the distal third of the leg [8]. For this reason, we choose a measurement site that was 5 cm proximal to the lateral malleolus as the measurement site.

Conventional high-frequency ultrasound with 10–14 MHz can also be used to identify the SN [11]–[15], but it does not reveal the investigator to evaluate the fine structure of the inner SN. Several reports have focused on the use of ultrasound to evaluate nerve decompression in diabetic patients [16], [17]. Studies focusing on monitoring the morphological changes of the cutaneous nerves of diabetics by US have not been previously performed.

The pathology of diabetic neuropathy is characterized by the progressive loss of myelinated nerve fiber. The nerve swelling is thought to be the result of a cascade of the following events: thickening of the vasculature, association of the basement membranes with perineurial and Schwann cells; accumulation of microfibrillar material in the vicinity of perineurial cells; and an increased diameter of endoneurial collagen fibrils [9], [18].We found that the MT was the most predictive parameter for diabetic cutaneous neuropathy, with a threshold values of 0.365, yielding a sensitivity of 90.3% and a specificity of 87.7%. Therefore, the MT may be considered an indicator for the level of swelling of the nerve fascicles. In the present study, CSA maybe the most commonly used for evaluation of nerve swelling among the US criterion for the evaluation of nerve swelling. Our study also demonstrated that CSA might be an important indicator for monitoring the morphological changes of diabetic cutaneous nerves. The optimal cut-off value for the CSA was 1.685 mm^2^, yielding an 82.2% specificity, and a relatively low sensitivity (64.5%). However, our CSA value in the control group was much lower than previous report (5.3±1.8 mm^2^) for 15 MHz US [19]. The possible explanation for this discrepancy is the 15 MHz probe used in the previous study, which may have made the observation and measurement of nerve borders difficult. In the study by Ding J [20], a quantitative analysis to determine the CSA of the SN in healthy Chinese subjects was performed by morphometric analysis during biopsy. The authors found that the CSA of the SN ranged from 0.60 mm^2^–1.23 mm^2^, which was similar to our measurements. Otherwise, the T/W ratio, which was used to detect the level of of SN deformation, was also used to evaluate the morphological changes in our study. This index was similar to the “flattening ratio”, which was initially emphasized by Buchberger et al. [21], [22]. There was a significant increase in the T/W ratio in the neuropathic group (0.59±0.09) compared to patients in the non-neuropathic group (0.56±0.10) and the control group (0.51±0.07). Moreover, assessment of the T/W ratio and the MT was first proposed as a means of examining nerve injuries. However, additional clinical studies with large sample sizes are required to establish the usefulness of these parameters.

The results of this study suggest that US may be a valuable tool for evaluating diabetic cutaneous nerve neuropathy. However, we believe that our study is only a preliminary report. The main limitation of our study is that we did not compare our sonographic findings to changes in histological appearance. Further studies are needed to overcome these limitations.

## References

[1] Papanas N, Giassakis G, Papatheodorou K, Papazoglou D, Monastiriotis C (2007). Sensitivity and specificity of a new indicator test (Neuropad) for the diagnosis of peripheral neuropathy in type 2 diabetes patients: a comparison with clinical examination and nerve conduction study.. Diabetes Complications.

[2] Hirai A, Yasuda H, Joko M, Maeda T, Kikkawa R (2000). Evaluation of diabetic neuropathy through the quantitation of cutaneous nerves.. J Neurol Sci.

[3] Meijer JW, Smit AJ, Sonderen EV, Groothoff JW, Eisma WH (2002). Symptom scoring systems to diagnose distal polyneuropathy in diabetes: the Diabetic Neuropathy Symptom score.. Diabet Med.

[4] Fioretti S, Scocco M, Ladislao L, Ghetti G, Rabini RA (2010). Identification of peripheral neuropathy in type-2 diabetic subjects by static posturography and linear discriminant analysis.. Gait Posture.

[5] Ziegler D (2005). Validation of a novel screening device (Neuroquick) for quantitative assessment of small nerve fiber dysfunction as an early feature of diabetic polyneuropathy.. Diabetes Care.

[6] Ugrenovic S, Vasovic L, Jovanovic I, Stefanovic N (2004). Peculiarities of the sural nerve complex morphologic types in human fetuses.. Surg Radiol Anat.

[7] Shankar N, Selvam RP, Dhanpal N, Reddy R, Alapati A (2010). Anatomical variations of the sural nerve in the leg: a fetal study.. Neurol India.

[8] Pyun SB, Kwon HK (2008). The effect of anatomical variation of the sural nerve on nerve conduction studies.. Am J Phys Med Rehabil.

[9] Yagihashi S, Yamagishi S, Wada R (2007). Pathology and pathogenetic mechanisms of diabetic neuropathy: correlation with clinical signs and symptoms.. Diabetes Res Clin Pract.

[10] Braune HJ (1999). Testing of the refractory period in sensory nerve fibres is the most sensitive method to assess beginning polyneuropathy in diabetics.. Electromyogr Clin Neurophysiol.

[11] Zhu J, Li D, Shao J, Hu B (2011). An ultrasound study of anatomic variants of the sural nerve.. Muscle Nerve.

[12] Ricci S, Moro L, Antonelli IR (2010). Ultrasound imaging of the sural nerve: ultrasound anatomy and rationale for investigation.. Eur J Vasc Endovasc Surg.

[13] Flavin R, Gibney RG, O’Rourke SK (2007). A clinical test to avoid sural nerve injuries in percutaneous Achilles tendon repairs.. Injury.

[14] Kamm CP, Scheidegger O, Rösler KM (2009). Ultrasound-guided needle positioning in sensory nerve conduction study of the sural nerve.. Clin Neurophysiol.

[15] Redborg KE, Sites BD, Chinn CD, Gallagher JD, Ball PA, Antonakakis JG (2009). Ultrasound improves the success rate of a sural nerve block at the ankle.. Reg Anesth Pain Med.

[16] Lee D, Dauphinée DM (2005). Morphological and functional changes in the diabetic peripheral nerve: using diagnostic ultrasound and neurosensory testing to select candidates for nerve decompression.. J Am Podiatr Med Assoc.

[17] Watanabe T, Ito H, Sekine A, Katano Y, Nishimura T (2010). Sonographic evaluation of the peripheral nerve in diabetic patients: the relationship between nerve conduction studies, echo intensity, and cross-sectional area.. J Ultrasound Med.

[18] Yasuda H, Terada M, Maeda K, Kogawa S, Sanada M (2003). Diabetic neuropathy and nerve regeneration.. Progress in Neurobiology.

[19] Cartwright MS, Passmore LV, Yoon JS, Brown ME, Caress JB (2008). Cross-sectional area reference values for nerve ultrasonography.. Muscle Nerve.

[20] Ding J, Jiang XM, Lin SH (2001). Morphometric studies on the myelinated fibre of healthy human sural nerve.. Chin J Neurol.

[21] Buchberger W, Schön G, Strasser K, Jungwirth W (1991). High-resolution ultrasonography of the carpal tunnel.. J Ultrasound Med.

[22] Visser LH, Smidt MH, Lee ML (2007). High-resolution sonography versus EMG in the diagnosis of carpal tunnel syndrome.. J Neurol Neurosurg Psychiatry.

